# Prediction of early treatment response to the combination therapy of TACE plus lenvatinib and anti-PD-1 antibody immunotherapy for unresectable hepatocellular carcinoma: Multicenter retrospective study

**DOI:** 10.3389/fimmu.2023.1109771

**Published:** 2023-02-17

**Authors:** Shuqun Li, Junyi Wu, Jiayi Wu, Yangkai Fu, Zhenxin Zeng, Yinan Li, Han Li, Weijia Liao, Maolin Yan

**Affiliations:** ^1^ Shengli Clinical Medical College of Fujian Medical University, Fuzhou, Fujian, China; ^2^ Department of Hepatobiliary Pancreatic Surgery, Affiliated Hospital of Guilin Medical University, Guilin, Guangxi, China; ^3^ Department of Hepatobiliary Pancreatic Surgery, Fujian Provincial Hospital, Fuzhou, Fujian, China

**Keywords:** unresectable hepatocellular carcinoma, TACE, levantinib, anti-PD-1 antibody, immunotherapy, nomogram

## Abstract

**Background and aim:**

The purpose of this study was to investigate and validate the efficacy of a nomogram model in predicting early objective response rate (ORR) in u-HCC patients receiving a combination of TACE, Lenvatinib, and anti-PD-1 antibody treatment after 3 months (triple therapy).

**Method:**

This study included 169 u-HCC cases from five different hospitals. As training cohorts (n = 102), cases from two major centers were used, and external validation cohorts (n = 67) were drawn from the other three centers. The clinical data and contrast-enhanced MRI characteristics of patients were included in this retrospective study. For evaluating MRI treatment responses, the modified revaluation criteria in solid tumors (mRECIST) were used. Univariate and multivariate logistic regression analyses were used to select relevant variables and develop a nomogram model. Our as-constructed nomogram was highly consistent and clinically useful, as confirmed by the calibration curve and decision curve analysis (DCA); an independent external cohort also calibrated the nomogram.

**Results:**

The ORR was 60.9% and the risk of early ORR was independently predicted by AFP, portal vein tumor thrombus (PVTT), tumor number, and size in both the training (C-index = 0.853) and test (C-index = 0.800) cohorts. The calibration curve revealed that the nomogram-predicted values were consistent with the actual response rates in both cohorts. Furthermore, DCA indicated that our developed nomogram performed well in clinical settings.

**Conclusion:**

The nomogram model accurately predicts early ORR achieved by triple therapy in u-HCC patients, which aids in individual decision-making and modifying additional therapies for u-HCC cases.

## 1 Introduction

Hepatocellular carcinoma (HCC) is one of the most commonly diagnosed cancers and the third leading cause of cancer-related deaths worldwide. However, over 1/2 of HCC cases are diagnosed at advanced stages, thus, depriving patients of undergoing surgical resection (1, 2). Moreover, there has been tremendous recent progress in managing u-HCC by enhancing anti-HCC systemic protocols for better prognosis (3, 4).

In 2007, sorafenib was the only approved therapeutic agent for treating uHCC. Thereafter, with the advent of new molecular-targeted agents, lenvatinib, in 2018, demonstrated a comparable therapeutic effect to sorafenib as a first-line treatment (5–7). Besides the introduction of monoclonal antibody (mAB) and tyrosine kinase inhibitors (TKIs) in systemic treatment, immune checkpoint blockers (ICBs)-based immunotherapy is now widely utilized in HCC cases (8–10). Additionally, the objective response rate (ORR) of recommended first-line therapies was low, as single-agent and dual therapies have limited efficacy (5, 6, 11, 12). To reduce tumor burden and improve u-HCC prognosis, various combination regimens, including systemic and locoregional therapies, are now used. Our previous study found that the ORR after triple therapy was 80.6% (TACE, lenvatinib, and Anti-PD-1 antibody immunotherapy) (13–17). Despite the triple therapy, several patients experienced disease progression. Thus, identifying the patients developing innate triple therapy resistance can help develop additional treatment protocols and avoid unnecessary financial strain.

Although several articles have predicted the prognosis of many local or systemic treatments, they have either focused on predicting overall survival (OS) and recurrence postoperatively or estimating alpha-fetoprotein (AFP) levels during the treatment, intraoperative conditions, and postoperative pathologies, thus, making prognosis determination difficult (18–22).

The purpose of this study was to identify predictors of early ORR based on simple variables that are easy to collect, as well as to develop and validate a simple, reproducible, and accurate nomogram in u-HCC patients undergoing triple therapy, which could aid clinical decision-making and provide individualized treatment for u-HCC patients.

## 2 Materials and methods

### 2.1 Patients and study design

This multicenter retrospective study included those u-HCC patients who received a combination of TACE, lenvatinib, and anti-PD-1 antibody therapy between October 28, 2018, and April 5, 2022, at five centers. The training cohort (n = 102) included patients from the hepatobiliary and pancreatic departments of Fujian Provincial Hospital and Guilin Medical University. The external validation cohort (n = 67) included patients from Zhangzhou Municipal Hospital of Fujian Province, Fujian Medical University Union Hospital, and the First Affiliated Hospital of Xiamen University. Model construction and assessment were completed based on the training and test sets, respectively.

We took approval from Institutional Review Boards (IRBs) from the five involved centers, and the study was carried out following the principle of the Declaration of Helsinki. The HCC diagnosis in all patients was based on non-invasive standards adopted *via* the American Association for the Study of Liver Disease (AASLD) and the European Association for the Study of the Liver (EASL). Tumor unresectability was determined by either the advanced disease stage or inadequate liver remnants following hepatectomy (<40% and <30% in liver cirrhosis and non-liver cirrhosis cases, respectively). Our study protocols gained approval from the Research Ethics Committee of Fujian Provincial Hospital, while informed consent was obtained from all patients before initiating the triple therapy.

The inclusion criteria were: 1) patients aged >18 years; 2) those with the Eastern Cooperative Oncology Group (ECOG) score of 0–2; 3) patients diagnosed with u-HCC one week before the treatment, and 4) those who did not receive any HCC-associated treatment (locoregional or systemic). The exclusion criteria were: 1) those with additional concurrent primary cancers; 2) contraindications to the triple therapy; 3) those without basic clinical or imaging data, and 4) treatment discontinuation.

### 2.2 TACE therapy

Our cases underwent conventional TACE (cTACE) therapy. Depending on the reserved liver function and tumor location, a 2.7 F microcatheter was injected *via* subsegmental or segmental feeding arteries. Chemoembolization was performed using intra-arterial pirarubicin (20–60 mg), oxaliplatin (200 mg), and lipiodol (5–20 ml), followed by injection of gelatin sponge particles until arterial flow was significantly reduced. The amount of emulsion injection was determined by measuring the tumor volume. Furthermore, TACE was repeated based on residual detection and follow-up examinations. Supportive care was provided when patients were unsuitable for receiving subsequent TACE therapy. Every TACE cycle was implemented *via* interventional radiologists who had >5 years of experience (23).

### 2.3 Lenvatinib treatment

Lenvatinib was administered at 8 mg/day and 12 mg/day for cases weighing <60 kg and those>weighing more than 60 kg, respectively. However, the dose was reduced if treatment-related adverse events (TRAEs) occurred. Lenvatinib was discontinued in patients with persistent grade 3/4 TRAEs following dose reduction until the alleviation and disappearance of TRAEs. Any cases not adhering to our treatment were eliminated.

### 2.4 Anti-PD-1 antibodies administration

Intravenous injection of anti-PD-1 antibodies was given: camrelizumab (200 mg), sintilimab (200 mg), or tislelizumab (200 mg) at 3-week intervals. In cases of severe TRAEs or disease progression, the drug was discontinued.

### 2.5 Follow-up

All patients were treated and monitored on a monthly basis. At the time of enrollment, the BCLC staging, physical examination, and laboratory investigations such as routine blood tests, liver function tests, coagulation function tests, alpha-fetoprotein (AFP) levels, abdominal contrast-enhanced MRI, and chest CT were all documented. The formula −0.085 × (albumin g/L) + 0.66 × log (bilirubin µmol/l) was applied in determining the albumin-bilirubin (ALBI) score: ALBI ≤ –2.60 was defined as low, and ALBI>-2.60 was designated as high (24). Two radiologists blinded to clinical information with 5–6 years of experience reviewed radiological images for assessing HCC imaging characteristics. However, any disagreement between them was settled down through mutual negotiation. In the case of >2 tumors, we defined it as multiple, and the largest one was analyzed; otherwise, it was categorized as single. We measured the longest diameter of the largest tumor (for multiple tumors) and the maximum cross-sectional diameter of the tumor.

mRECIST criteria were applied in assessing the tumor response. Our study’s outcome measurement was ORR obtained in 3 months, including partial (PR, 30% decrease in arterial-enhancing lesions) and complete responses (CR, arterial-enhancing lesion disappearance). The National Cancer Institute Common Terminology Criteria for Adverse Events v4.0 was employed for assessing the AEs.

### 2.6 Statistical analysis

Descriptive statistics were utilized to summarize basic radiological data regarding tumor responses. Data were represented by frequency, mean ± SD, or median, and 95% confidence interval (CI). Continuous variables were compared by the Mann-Whitney U test, whereas categorical variables were analyzed by Fisher’s exact test or Pearson’s chi-square test. Differences in clinical characteristics were assessed by univariate regression analysis, while significant variables were incorporated into multivariate regression analysis with a binary logistic regression model for identifying ORR-related predictors. SPSS 26.0 software (SPSS Inc., Chicago, IL) was used for statistical analysis.

By R package (version 3.2.0) “rms” package, a predictive nomogram was established based on several independent factors assessed by multivariate analysis. After that, this work determined the concordance index (C-index), while our nomogram prediction accuracy was evaluated by drawing calibration plots. Furthermore, the model was validated by 1000 bootstraps for quantifying the overfitting modeling strategy while predicting model prediction accuracy. In our study, each statistical test was two-sided, with *p*<0.05 indicating statistical significance.

## 3 Results

### 3.1 Baseline features

Patients were followed up for 4 months. A total of 169 patients who received a combination of TACE, lenvatinib, and anti-PD-1 immunotherapy were included. Best responses were 24 CR, 79 PR, 42 SD, and 24 PD respectively. The 3 months ORR of the entire cohort was 60.9%, comprising 147 males and 22 females, respectively. Most of the patients <65 years. In total, 133 (78.7%) cases showed an ECOG score of 0. Most cases developed hepatitis B. A total of 167 patients had Child-Pugh class A scores, while most cases were staged as either BCLC stage B (61, 36.1%) or C (93, 55.3%). Seventy-two patients (42.6%) had portal vein tumor thrombus (PVTT), while we had 100 AFP-positive cases (59.1%). Altogether 127 (75.1%) cases had multiple tumor numbers, whereas 144 (85.2%) patients reported the biggest tumor diameter of>5cm. **Table 1** presents detailed information regarding the patient’s general characteristics.

**Table 1:** Main baseline demographic and clinical characteristics of patients in the training and validation cohorts (n=169).

		Training set (n=102)	Validation set (n=67)	Standardized differences	P
Sex	F	12 (11.8%)	10 (14.9%)	0.093	0.55
	M	90 (88.2%)	57 (85.1%)		
age	<=65	78 (76.5%)	48 (71.6%)	-0.110	0.481
	>65	24 (23.5%)	19 (28.4%)		
BCLC	A	10 (9.8%)	5 (7.5%)	0.368	0.76
	B	38 (37.3%)	23 (34.3%)		
	C	54 (52.9%)	39 (58.2%)		
Child-Pugh	A	101 (99%)	66 (98.5%)	-0.046	1
	B	1 (1%)	1 (1.5%)		
ECOG-PS	0	82 (82.8%)	51 (78.5%)	-0.111	0.485
	1	17 (17.2%)	14 (21.5%)		
AFP	<=20	42 (41.2%)	27 (40.3%)	-0.018	0.91
	>20	60 (58.8%)	40 (59.7%)		
HBV	No	7 (6.9%)	6 (9%)	0.078	0.618
	Yes	95 (93.1%)	61 (91%)		
Tumor size	<5	16 (15.7%)	9 (13.4%)	0.367	0.92
	5~10	47 (46.1%)	32 (47.8%)		
	>10	39 (38.2%)	26 (38.8%)		
Tumor no.	Single	24 (23.5%)	18 (26.9%)	0.077	0.623
	Multiple	78 (76.5%)	49 (73.1%)		
Anti-PD-1 antibodies	Camrelizumab	56 (50.0%)	31 (46.3%)	0.693	0.372
	Sintilimab	27 (26.5%)	19 (28.4%)		
	Tislelizumab	19 (23.5%)	17 (25.3%)		
PVTT	No	58 (56.9%)	39 (58.2%)	0.027	0.863
	Yes	44 (43.1%)	28 (41.8%)		
ALBI	High	57 (58.2%)	36 (56.3%)	-0.039	0.81
	Low	41 (41.8%)	28 (43.8%)		
ALT	0	50 (51%)	35 (54.7%)	0.074	0.648
	1	48 (49%)	29(45.3%)		
AST	0	34 (34.7%)	24 (37.5%)	0.058	0.716
	1	64 (65.3%)	40 (62.5%)		
WBC		6.64 ± 2.41	6.99 ± 2.5	0.143	0.369
RBC		4.71 ± 0.87	4.65 ± 0.93	-0.067	0.697
Hb		139.36 ± 23.77	138.36 ± 24.3	-0.042	0.796
PLT		206.39 ± 106.13	218.81 ± 102.2	0.119	0.461
Neu		4.36 ± 2.12	4.59 ± 2.25	0.105	0.512
Mono		0.51 ± 0.26	0.54 ± 0.25	0.118	0.380
Lym		1.55 ± 0.7	1.6 ± 0.7	0.071	0.687
GGT		169.37 ± 141.34	160.43 ± 148.8	-0.062	0.694
AKP		135.89 ± 81.38	140.03 ± 92.86	0.047	0.765
PT^©^		12.04 ± 1	12.07 ± 1.01	0.030	0.881
INR^©^		1.05 ± 0.09	1.06 ± 0.08	0.117	0.831
APTT		27.96 ± 3.41	28.11 ± 3.74	0.042	0.804
TT		17.59 ± 1.55	17.45 ± 1.51	-0.091	0.576
FIB		3.19 ± 0.98	3.29 ± 1.03	0.099	0.513

BCLC, Barcelona Clinic for Liver Cancer; HBV, hepatitis B virus; AFP, α-fetoprotein; PVTT, Portal vein tumor thrombus; ALT: alanine transaminase; AST: aspartate transaminase; WBC, white blood cell count; RBC, red blood cell count; Hb, hemoglobin; PLT, platelet; Neu, Neutrophils; Mono, Monocyte macrophages; Lym, lymph node cell node cells; GGT, gamma-glutamyl transferase; AKP, Alkaline phosphatase; PT, Prothrombin time; I NR, International normalized Ratio; APTT, Activated partial coagulation time; TT, Prothrombin time; FIB, Fibrinogen.

### 3.2 Adverse events

**Table 2** depicts two cohorts of TRAEs. At least one TRAE was reported by 142 patients (84%). Abdominal liver function (62.3%), fever(30.8%), hypertension (27.2%), and fatigue (23.7%) were the most common TRAEs. TRAEs were minor to moderate in most patients, and there was no toxicity-induced death. Seven patients (4.7%) experienced grade 4/5 TRAEs, which were alleviated by a lower dose of lenvatinib.

**Table 2:** Treatment-related adverse events.

Adverse Events	Any grade n(%)	Grade1-2 n(%)	Grade 3 n(%)	Grade 4 n(%)
Total	142 (84.0)	103 (60.9)	32 (18.9)	7 (4.1)
Fatigue	40 (23.7)	34 (20.1)	6 (3.6)	–
Decreased appetite	39 (23.1)	30 (17.8)	9 (5.3)	–
Fever	52 (30.8)	50 (29.6)	2 (1.2)	–
Nausea	30 (17.8)	24 (14.2)	6 (3.6)	–
Vomiting	18 (10.7)	15 (8.9)	3 (1.8)	–
Abdominal pain	20 (11.8)	18 (10.7)	2 (1.1)	–
Hand-foot syndrome	24 (14.2)	19 (11.2)	4 (2.4)	1 (0.6)
Diarrhea	21 (12.4)	21 (12.4)	–	–
Hypertension	46 (27.2)	36 (21.3)	10 (5.9)	–
Proteinuria	18 (10.7)	12 (7.1)	5 (2.9)	1 (0.6)
Skin rash	19 (11.2)	13 (7.7)	4 (2.4)	2 (1.1)
Thrombocytopenia	24 (14.2)	24 (14.2)	–	–
Hypothyroidism	28 (16.6)	22 (13.0)	6 (3.6)	–
Abnormal liver function	105 (62.3)	86 (50.9)	16 (9.5)	3 (1.9)

### 3.3 Independent factors associated with ORR and predictive nomogram construction

Univariate analysis identified age >65 years *(p* = 0.035), AFP >20 ng/mL (*p* = 0.035), and presence of PVTT (*p* = 0.027), multiple tumor numbers (*p* = 0.024), tumor size >5cm (*p* = 0.022) and 10cm (*p* = 0.023), and gamma-glutamyl transferase (GGT) as factors independently predicting prognosis and were further used to construct a model (**Table 3**). The multivariate regression analysis showed that these five factors affected early ORR and are enumerated in **Table 4**.

**Table 3:** Univariate Cox regression analyses for early ORR of u-HCC patients in the training cohor.

		ORR negative	ORR positive	T/Z/c^2^	P
Sex	F	6(15%)	6(9.7%)	0.664	0.415
	M	34(85%)	56(90.3%)		
age	<=65	35(87.5%)	43(69.4%)	4.449	**0.035**
	>65	5(12.5%)	19(30.6%)		
BCLC	A	2(5%)	8(12.9%)	4.286	0.117
	B	12(30%)	26(41.9%)		
	C	26(65%)	28(45.2%)		
Child-Pugh	A	40(100%)	61(98.4%)	–	1△
	B	0(0%)	1(1.6%)		
PS	0	30(76.9%)	52(86.7%)	1.578	0.209
	1	9(23.1%)	8(13.3%)		
HBV	No	3(7.5%)	4(6.5%)	–	1△
	Yes	37(92.5%)	58(93.5%)		
Anti-PD-1 antibodies	Camrelizumab	22(55.0%)	34(54.8%)	1.835	0.364
	Sintilimab	10(25.0%)	17(27.4%)		
	Tislelizumab	8(20.0%)	11(17.8%)		
PVTT	No	13(32.5%)	45(72.6%)	15.924	**<0.001**
	Yes	27(67.5%)	17(27.4%)		
ALBI	High	23(59%)	34(57.6%)	0.018	0.895
	Low	16(41%)	25(42.4%)		
ALT	0	23(59%)	27(45.8%)	1.64	0.2
	1	16(41%)	32(54.2%)		
AST	0	13(33.3%)	21(35.6%)	0.053	0.818
	1	26(66.7%)	38(64.4%)		
AFP	<=20	6(15.0%)	36(58.1%)	18.616	**<0.001**
	>20	34(85.0%)	26(41.9%)		
Tumor cm	<5	3(7.5%)	13(21.0%)	16.637	**0.001**
	5~10	12(30.0%)	35(56.5%)		
	>10	25(62.5%)	14(22.6%)		
Tumor No	single	5(12.5%)	19(30.6%)	4.449	**0.035**
	multiple	35(87.5%)	43(69.4%)		
WBC		6.3(5.27,7.8)	6.4(4.9,8.1)	-0.091	0.928
RBC		4.64(4.32,4.98)	4.67(4.08,5.38)	-0.214	0.83
Hb		138 ± 20.2	140.25 ± 25.99	-0.458	0.648
PLT		191(126,245)	185(137,251)	-0.171	0.865
Neu		3.8(2.7,5.2)	4(2.6,5.6)	-0.192	0.847
mono		0.44(0.33,0.63)	0.42(0.32,0.67)	-0.584	0.559
Lym		1.6(1.1,1.9)	1.5(0.99,2)	-0.432	0.666
GGT		179(87.5,296.8)	119.5(58,167)	-2.183	**0.029**
AKP		120(88,158)	111(88.8,145)	-0.925	0.355
PT		12.13 ± 1.05	11.98 ± 0.97	0.701	0.485
INR		1.06 ± 0.09	1.05 ± 0.08	0.371	0.712
APTT		27.3(25.58,29.18)	27.3(25.8,28.95)	-0.137	0.891
TT		17.6(16.68,18.33)	17.4(16.35,18.5)	-0.578	0.564
FIB		3.04(2.49,3.98)	2.95(2.45,3.78)	-0.368	0.713

Bold values is to highlight the meaningful p-values.

**Table 4:** Multivariate Cox regression analyses for early ORR of u-HCC patients in the training cohort.

	B	SE	Wald	P	OR	95%CI
Tumorsize<5	1.955	0.856	5.211	0.022	7.062	1.318-37.826
Tumorsize5-10	1.403	0.617	5.174	0.023	4.067	1.214-13.626
Tumorsize>10	0				1	
tumorNo single/multiple	1.609	0.714	5.079	0.024	5.000	1.233-20.267
AFP <=20/>20	1.256	0.595	4.457	0.035	3.511	1.094-11.263
PVTT No/Yes	1.218	0.55	4.915	0.027	3.382	1.152-9.932
Age<=65/>65	-1.004	0.689	2.126	0.145	0.366	0.095-1.413
GGT	-0.001	0.002	0.415	0.519	0.999	0.995-1.002
Constants	-0.881	0.813	1.174	0.279	0.414	

**Figure 1:**
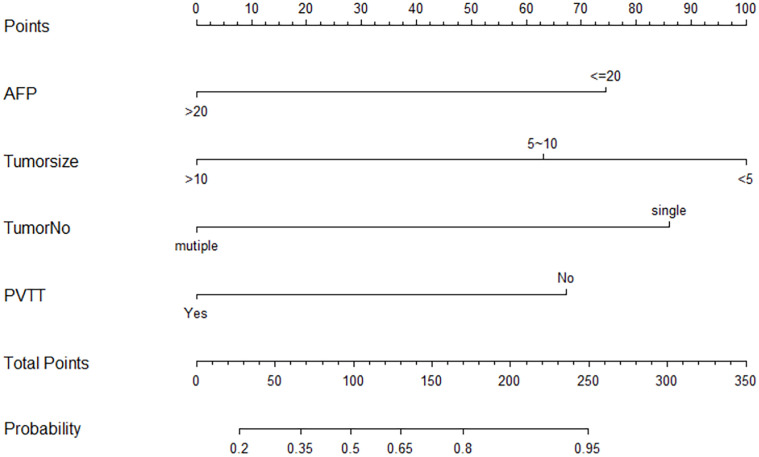
Nomogram to predict the early ORR for patients in the training cohort.

Following that, we aimed to develop a nomogram for predicting the ORR of triple therapy using the four independent factors mentioned above that were identified through univariate and multivariate regression analyses on the training cohort (**Figure 1**). The factors included in our nomogram were given the weighted point number. At the same time, the sum of every case was associated with the specific predicted ORR rate. Moreover, the optimal cut-off point of the nomogram was 100, the increase in total points predicted an increased ORR.

### 3.4 Model performance and validation

**Figure 2:**
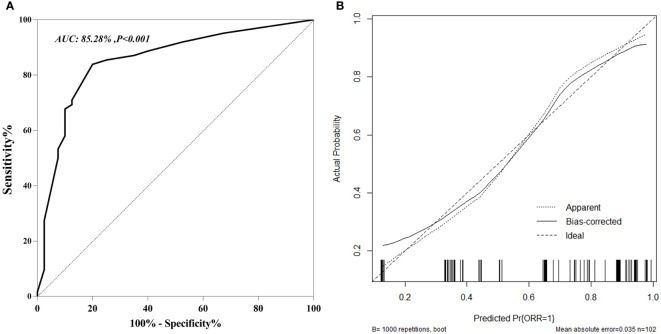
ROC curves **(A)** and Calibration curves **(B)** for predicting and validation early ORR in u-HCC patients in training and cohort.

**Figure 3:**
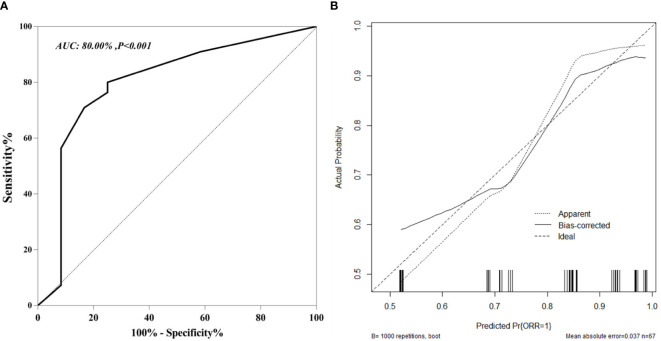
ROC curves **(A)** and Calibration curves **(B)** for predicting and validation early ORR in u-HCC patients in external validation cohort.

**Figure 4:**
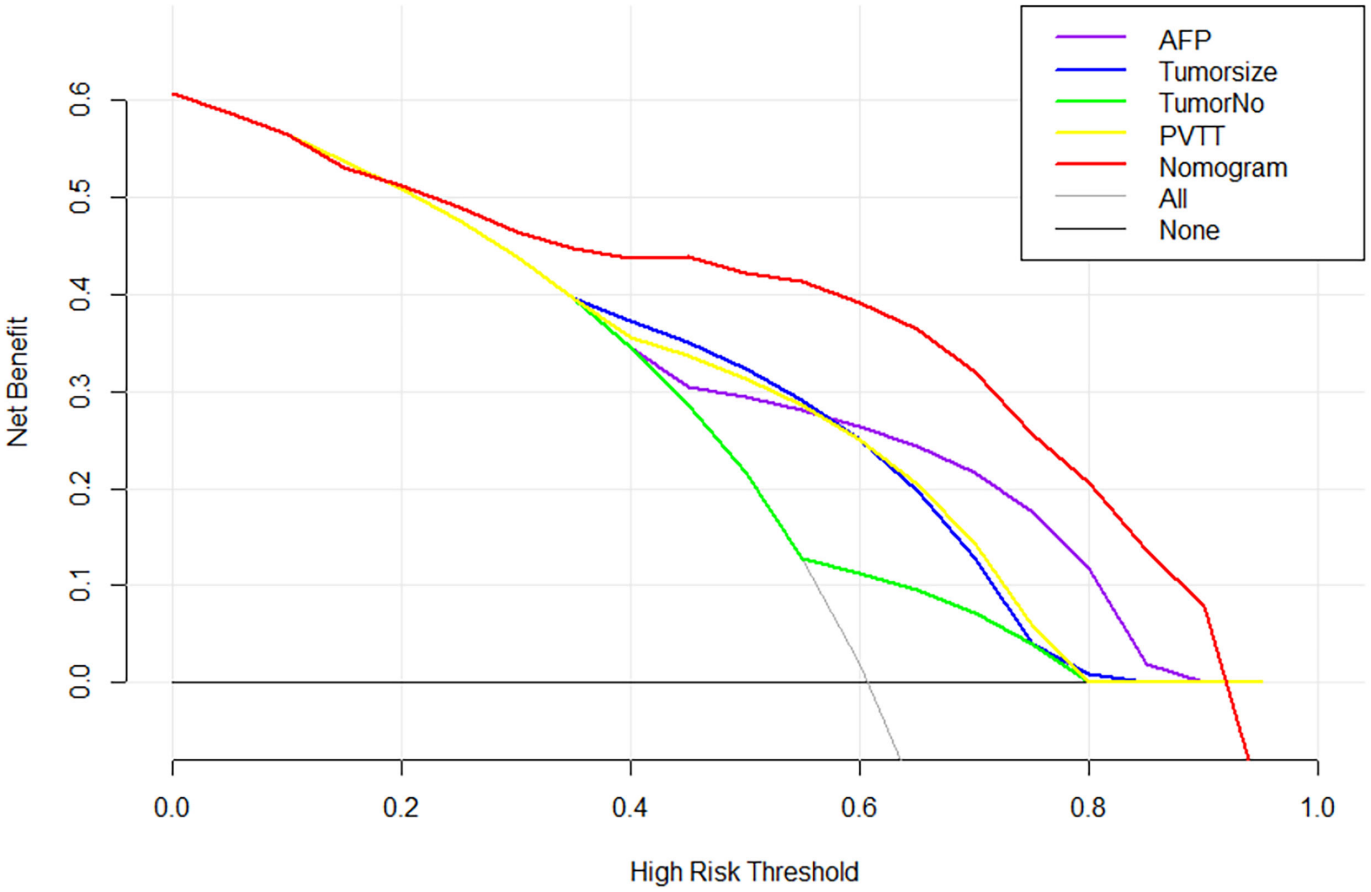
Decision curve analysis for early ORR in the training cohort. Black line: All patients dead. Gray line: None patients dead. Red line: Model of nomogram.

The nomogram C-index was 85.28% (95% CI, 77.50%-93.07%) for the training cohort. Moreover, the plotted calibration curves were near the ideal 45° line, indicating that the nomogram-predicted ORR was highly consistent with real measurements at each time point (**Figure 2**). Concerning the external test set, our constructed nomogram was accurate in ORR prediction. The C-index was 80.00% (95% CI, 63.52%−87.83%, **Figure 3**). DCA also showed that our nomogram was effective for ORR prediction after comparison with treated and untreated cases as the training and test sets, respectively (**Figure 4**).

## 4 Discussion

In the current study, triple therapy was administered to 169 u-HCC patients who were followed up on for ≥4 months across multiple centers. The patients in BCLC stage C had 55.3%, proving that triple therapy can be used clinically for advanced-stage HCC patients. The early ORR in all u-HCC cohorts was 60.9%. This value, however, is higher than the findings in some first-line and combination therapies for u-HCC cases, and it has the potential to become the standard therapy for u-HCC patients. The ORR of the sorafenib group in the SHARP trial was only 2% (5). Additionally, according to a phase-III REFLECT trial, lenvatinib attained an ORR of 24.1%, the highest ORR obtained in monotherapy (6). Besides, the ORR of the atezolizumab and bevacizumab therapy for advanced HCC was 33.2% according to the IMbrave150 trial (9), while the dual-therapy application resulted in an ORR of 13.6%-46% in u-HCC cases (25, 26). The occurrence of high ORR in the triple treatment might be due to the following reasons: 1) TACE direct impairs tumors while reducing the tumor burden, thus, inducing a hypoxic and ischemic microenvironment and causing tumor-specific antigen production as well as necrosis. 2) Lenvatinib enhances infiltrating capacity of immune and effector T-cells in the tumor microenvironment, improves immune status, prevents T-cell exhaustion, and suppresses immunosuppressive cell activity. This further reduces Treg differentiation and PD-L1 content in the tumor, thereby reducing TGF-ß signaling and FGFR3 inhibition for improving the anti-PD-1 therapeutic effect (27–29).

These findings revealed that AFP, PVTT, tumor number, and size were the independent factors that predicted an early ORR. Therefore, we developed a nomogram based on several factors to predict early ORR to triple therapy. The C-index calculation and calibration plot drawing of the nomogram demonstrated that our as-constructed nomogram performed well in evaluating early ORR. An external cohort also calibrated the nomogram, with a high concordance index of 0.800. AFP ≥20ng/mL was inversely correlated with early ORR in u-HCC patients in our model, as was observed in many previous studies (30, 31). AFP is a widely used prognostic biomarker used for numerous HCC prediction models. It promotes tumor development by inhibiting apoptosis and blocking the anti-tumor effect because of its inhibitory properties on T lymphocyte growth, dendritic cell (DC) differentiation, and natural killer (NK) cell activities as enhancing effects on suppressor T cell activity. Furthermore, many studies have indicated the correlation of AFP with increased VEGF expression (32, 33). PVTT is the most common form of macrovascular invasion, with incidence ranging from 44.0% to 62.2% in HCC cases (34), which might block anti-HCC treatment and depict a dismal prognostic outcome. Llovet et al. examined the natural history of HCC cases with PVTT and stated that the median survival time (MST) for untreated cases was 2.7 months (35). Recently, MahringerKunz et al. examined 1317 HCC cases with PVTT and revealed MST as 7.2 months, which had decreased when compared to cases having no PVTT (35.7 months, *p*<0.001) (36). Our retrospective study included 42.6% of PVTT patients and yielded a very high ORR, demonstrating the efficacy of the triple treatment in u-HCC patients. In agreement with previous studies, our findings revealed that PVTT was an independent predictor of prognosis, which was related to early ORR of u-HCC cases. Patients with PVTT have a lower early ORR than those without PVTT, which could be due to a change in hepatic blood supply due to the hypoxic microenvironment and increased angiogenic factor levels after an ischemic liver injury (29, 37). Tumor size and number are the potential prognostic factors for HCC cases irrespective of either locoregional or systemic treatment initiation (38, 39). Similarly, these two factors were also the key independent factors depicting early tumor response to triple therapy. Hence, patients with large tumor diameters and multiple tumors display worse prognoses compared to patients with small tumor diameters and single tumors. Additionally, the tumor size might be associated with the prognostic outcome with respect to other unfavorable risk factors like nutritional status, tumor-related microenvironment, vascular invasion, genetic history, and lower differentiation levels; a tumor’s aggressive behavior can also be a key indicator for developing malignancy (40, 41).

Several studies have constructed prediction models to investigate the prognostic factors for some locoregional and systemic therapies. Zhang et al. established a prognosis nomogram for HCC cases with portal vein metastasis who received TACE and sorafenib therapy and showed that PVTT, ALBI, and tumor size are key factors associated with OS (20). Scheiner et al. discovered that serum AFP and C-reactive protein (CRP) were independently related to poor OS among HCC cases receiving ICB treatment. Thus, developed CRAFITY scoring for predicting the prognosis of immunotherapy-treated HCC cases (42). Ours is the first study to build an early ORR model and validate triple therapy in patients with u-HCC. Furthermore, results from multiple-center cohorts were extracted and calibrated by an external set, lending greater confidence to our model. Furthermore, the prognostic factors proposed by our study are typically discovered during the practice work-up for HCC cases and will not incur additional costs. Furthermore, our predictive model, which was developed using common clinical and radiological baseline characteristics, is suitable for clinical use. Lastly, our study’s most important strength is that we can predict an early ORR before the treatment commences. Thus, our nomogram constructed by routine factors to predict ORR might help select cases that can benefit from triple therapy, assess unsuitable cases for triple therapy, and provide individualized therapy in u-HCC patients.

As this study was retrospective, it negatively affected our results. Despite being based on a large, multicenter study population, prospective clinical and large-scale studies need to confirm this nomogram model. Furthermore, hepatitis B virus (HBV) is the leading cause of HCC in the Chinese population. It may exhibit distinct tumor features when compared to other causes, such as alcohol use or hepatitis C virus (HCV). TACE procedure physicians have varying experiences from different centers, which may result in heterogeneity in TACE therapy. In the current study, the anti-PD-1 strategy was not the same. Because of clinically relevant differences in anti-PD-1 antibody treatments across studies, the anti-PD-1 strategy used in our study did not show much improvement. Regardless of the above limitations, our constructed nomogram exhibited high discriminating and calibrating capacities in predicting early ORR for u-HCC cases treated with triple therapy. Thus, by focusing on predicting the early ORR of the treatment, the differing ORR rates might lead to differences in the accuracy of the prediction models.

## 5 Conclusion

We constructed a nomogram for predicting early ORR for u-HCC cases receiving a combination of TACE, lenvatinib, and anti-PD-1 antibody immunotherapy. We have also provided reliable clinical data, specifically a feasible non-invasive method, for predicting ORR in u-HCC patients to promote patient-physician communication, appropriate treatment selection, and decision-making *via* personalized tumor response data.

## Data availability statement

The original contributions presented in the study are included in the article/supplementary material. Further inquiries can be directed to the corresponding author.

## Author contributions

All authors contributed to the study conception and design. Material preparation, data collection and analysis were performed by SL, JiW, JuW, YF, ZZ, YL, HL. The first draft of the manuscript was written by SL and all authors commented on previous versions of the manuscript. All authors contributed to the article and approved the submitted version.

## References

[1] Ferlay J Colombet M Soerjomataram I Mathers C Parkin DM Piñeros M. Estimating the global cancer incidence and mortality in 2018: Globocan sources and methods. Int J Cancer (2019) 144(8):1941–53. doi: 10.1002/ijc.31937 30350310

[2] Forner A Reig M Bruix J. Hepatocellular carcinoma. Lancet (2018) 391(10127):1301–14. doi: 10.1016/s0140-6736(18)30010-2 29307467

[3] O'Leary C Mahler M Soulen MC. Curative-intent therapies in localized hepatocellular carcinoma. Curr Treat Options Oncol (2020) 21(4):31. doi: 10.1007/s11864-020-0725-3 32193784

[4] Roderburg C Özdirik B Wree A Demir M Tacke F. Systemic treatment of hepatocellular carcinoma: From sorafenib to combination therapies. Hepat Oncol (2020) 7(2):Hep20. doi: 10.2217/hep-2020-0004 32647565 PMC7338920

[5] Llovet JM Ricci S Mazzaferro V Hilgard P Gane E Blanc JF. Sorafenib in advanced hepatocellular carcinoma. N Engl J Med (2008) 359(4):378–90. doi: 10.1056/NEJMoa0708857 18650514

[6] Kudo M Finn RS Qin S Han KH Ikeda K Piscaglia F. Lenvatinib versus sorafenib in first-line treatment of patients with unresectable hepatocellular carcinoma: A randomised phase 3 non-inferiority trial. Lancet (2018) 391(10126):1163–73. doi: 10.1016/s0140-6736(18)30207-1 29433850

[7] Lencioni R Llovet JM. Modified recist (Mrecist) assessment for hepatocellular carcinoma. Semin Liver Dis (2010) 30(1):52–60. doi: 10.1055/s-0030-1247132 20175033 PMC12268942

[8] Cheng H Sun G Chen H Li Y Han Z Li Y. Trends in the treatment of advanced hepatocellular carcinoma: Immune checkpoint blockade immunotherapy and related combination therapies. Am J Cancer Res (2019) 9(8):1536–45. PMC6726979 31497341

[9] Finn RS Qin S Ikeda M Galle PR Ducreux M Kim TY. Atezolizumab plus bevacizumab in unresectable hepatocellular carcinoma. N Engl J Med (2020) 382(20):1894–905. doi: 10.1056/NEJMoa1915745 32402160

[10] Ren Z Xu J Bai Y Xu A Cang S Du C. Sintilimab plus a bevacizumab biosimilar (Ibi305) versus sorafenib in unresectable hepatocellular carcinoma (Orient-32): A randomised, open-label, phase 2-3 study. Lancet Oncol (2021) 22(7):977–90. doi: 10.1016/s1470-2045(21)00252-7 34143971

[11] Lee M Ryoo BY Hsu CH Numata K Stein S Verret W. Randomised efficacy and safety results for atezolizumab (Atezo)+ bevacizumab (Bev) in patients (Pts) with previously untreated, unresectable hepatocellular carcinoma (Hcc). Ann Oncol (2019) 30(S5):v875. doi: 10.1093/annonc/mdz394.030

[12] Cheng AL Kang YK Chen Z Tsao CJ Qin S Kim JS. Efficacy and safety of sorafenib in patients in the Asia-pacific region with advanced hepatocellular carcinoma: A phase iii randomised, double-blind, placebo-controlled trial. Lancet Oncol (2009) 10(1):25–34. doi: 10.1016/s1470-2045(08)70285-7 19095497

[13] Wu JY Yin ZY Bai YN Chen YF Zhou SQ Wang SJ. Lenvatinib combined with anti-Pd-1 antibodies plus transcatheter arterial chemoembolization for unresectable hepatocellular carcinoma: A multicenter retrospective study. J Hepatocell Carcinoma (2021) 8:1233–40. doi: 10.2147/jhc.S332420 PMC8502053 34676181

[14] Sun B Zhang L Sun T Ren Y Cao Y Zhang W. Safety and efficacy of lenvatinib combined with camrelizumab plus transcatheter arterial chemoembolization for unresectable hepatocellular carcinoma: A two-center retrospective study. Front Oncol (2022) 12:982948. doi: 10.3389/fonc.2022.982948 36172158 PMC9511022

[15] Teng Y Ding X Li W Sun W Chen J. A retrospective study on therapeutic efficacy of transarterial chemoembolization combined with immune checkpoint inhibitors plus lenvatinib in patients with unresectable hepatocellular carcinoma. Technol Cancer Res Treat (2022) 21:15330338221075174. doi: 10.1177/15330338221075174 35313780 PMC8943530

[16] Chen S Wu Z Shi F Mai Q Wang L Wang F. Lenvatinib plus tace with or without pembrolizumab for the treatment of initially unresectable hepatocellular carcinoma harbouring pd-L1 expression: A retrospective study. J Cancer Res Clin Oncol (2022) 148(8):2115–25. doi: 10.1007/s00432-021-03767-4 PMC9293824 34453221

[17] Li X Fu Z Chen X Cao K Zhong J Liu L. Efficacy and safety of lenvatinib combined with pd-1 inhibitors plus tace for unresectable hepatocellular carcinoma patients in China real-world. Front Oncol (2022) 12:950266. doi: 10.3389/fonc.2022.950266 35860582 PMC9289205

[18] Brunetti O Gnoni A Licchetta A Longo V Calabrese A Argentiero A. Predictive and prognostic factors in hcc patients treated with sorafenib. Med (Kaunas) (2019) 55(10). doi: 10.3390/medicina55100707 PMC6843290 31640191

[19] Wang Q Xia D Bai W Wang E Sun J Huang M. Development of a prognostic score for recommended tace candidates with hepatocellular carcinoma: A multicentre observational study. J Hepatol (2019) 70(5):893–903. doi: 10.1016/j.jhep.2019.01.013 30660709

[20] Zhang L Sun JH Hou ZH Zhong BY Yang MJ Zhou GH. Prognosis nomogram for hepatocellular carcinoma patients with portal vein invasion undergoing transarterial chemoembolization plus sorafenib treatment: A retrospective multicentre study. Cardiovasc Intervent Radiol (2021) 44(1):63–72. doi: 10.1007/s00270-020-02579-2 32965582

[21] Sun X Mei J Lin W Yang Z Peng W Chen J. Reductions in afp and pivka-ii can predict the efficiency of anti-Pd-1 immunotherapy in hcc patients. BMC Cancer (2021) 21(1):775. doi: 10.1186/s12885-021-08428-w 34218801 PMC8254996

[22] Liu B Shang X Shi JY Cui GZ Li X Wang NY. Early alpha-fetoprotein response is associated with survival in patients with hbv-related hepatocellular carcinoma receiving lenvatinib. Front Oncol (2022) 12:807189. doi: 10.3389/fonc.2022.807189 35251977 PMC8893311

[23] Varghese J Kedarisetty C Venkataraman J Srinivasan V Deepashree T Uthappa M. Combination of tace and sorafenib improves outcomes in bclc stages B/C of hepatocellular carcinoma: A single centre experience. Ann Hepatol (2017) 16(2):247–54. doi: 10.5604/16652681.1231583 28233748

[24] Johnson PJ Berhane S Kagebayashi C Satomura S Teng M Reeves HL. Assessment of liver function in patients with hepatocellular carcinoma: A new evidence-based approach-the albi grade. J Clin Oncol (2015) 33(6):550–8. doi: 10.1200/jco.2014.57.9151 PMC4322258 25512453

[25] Luo XY Wu KM He XX. Advances in drug development for hepatocellular carcinoma: Clinical trials and potential therapeutic targets. J Exp Clin Cancer Res (2021) 40(1):172. doi: 10.1186/s13046-021-01968-w 34006331 PMC8130401

[26] Kudo M. Systemic therapy for hepatocellular carcinoma: Latest advances. Cancers (Basel) (2018) 10(11). doi: 10.3390/cancers10110412 PMC6266463 30380773

[27] Yang F Xu GL Huang JT Yin Y Xiang W Zhong BY. Transarterial chemoembolization combined with immune checkpoint inhibitors and tyrosine kinase inhibitors for unresectable hepatocellular carcinoma: Efficacy and systemic immune response. Front Immunol (2022) 13:847601. doi: 10.3389/fimmu.2022.847601 35300339 PMC8922415

[28] Xu ZN Huang JJ Zhou J Huang WS Guo YJ Cai MY. Efficacy and safety of anti-Pd-1 monoclonal antibody in advanced hepatocellular carcinoma after tace combined with tki therapy. Zhonghua Nei Ke Za Zhi (2021) 60(7):630–6. doi: 10.3760/cma.j.cn112138-20200928-00841 34619840

[29] Ding X Sun W Li W Shen Y Guo X Teng Y. Transarterial chemoembolization plus lenvatinib versus transarterial chemoembolization plus sorafenib as first-line treatment for hepatocellular carcinoma with portal vein tumor thrombus: A prospective randomized study. Cancer (2021) 127(20):3782–93. doi: 10.1002/cncr.33677 34237154

[30] Labeur TA Berhane S Edeline J Blanc JF Bettinger D Meyer T. Improved survival prediction and comparison of prognostic models for patients with hepatocellular carcinoma treated with sorafenib. Liver Int (2020) 40(1):215–28. doi: 10.1111/liv.14270 PMC6973249 31579990

[31] Pallozzi M Di Tommaso N Maccauro V Santopaolo F Gasbarrini A Ponziani FR. Non-invasive biomarkers for immunotherapy in patients with hepatocellular carcinoma: Current knowledge and future perspectives. Cancers (Basel) (2022) 14(19). doi: 10.3390/cancers14194631 PMC9559710 36230554

[32] Zheng Y Zhu M Li M. Effects of alpha-fetoprotein on the occurrence and progression of hepatocellular carcinoma. J Cancer Res Clin Oncol (2020) 146(10):2439–46. doi: 10.1007/s00432-020-03331-6 PMC11804406 32725355

[33] Cammarota A Zanuso V Pressiani T Personeni N Rimassa L. Assessment and monitoring of response to systemic treatment in advanced hepatocellular carcinoma: Current insights. J Hepatocell Carcinoma (2022) 9:1011–27. doi: 10.2147/jhc.S268293 PMC9482774 36128575

[34] Zhang ZM Lai EC Zhang C Yu HW Liu Z Wan BJ. The strategies for treating primary hepatocellular carcinoma with portal vein tumor thrombus. Int J Surg (2015) 20:8–16. doi: 10.1016/j.ijsu.2015.05.009 26026424

[35] Llovet JM Bustamante J Castells A Vilana R Ayuso Mdel C Sala M. Natural history of untreated nonsurgical hepatocellular carcinoma: Rationale for the design and evaluation of therapeutic trials. Hepatology (1999) 29(1):62–7. doi: 10.1002/hep.510290145 9862851

[36] Mähringer-Kunz A Steinle V Düber C Weinmann A Koch S Schmidtmann I. Extent of portal vein tumour thrombosis in patients with hepatocellular carcinoma: The more, the worse? Liver Int (2019) 39(2):324–31. doi: 10.1111/liv.13988 30318826

[37] Khan AR Wei X Xu X. Portal vein tumor thrombosis and hepatocellular carcinoma - the changing tides. J Hepatocell Carcinoma (2021) 8:1089–115. doi: 10.2147/jhc.S318070 PMC8434852 34522691

[38] Tsilimigras DI Moris D Hyer JM Bagante F Sahara K Moro A. Hepatocellular carcinoma tumour burden score to stratify prognosis after resection. Br J Surg (2020) 107(7):854–64. doi: 10.1002/bjs.11464 32057105

[39] Tsilimigras DI Pawlik TM. Tumour burden score: An authentic, easy-to-Use prognostic marker for hepatocellular carcinoma. Br J Surg (2020) 107(12):e626. doi: 10.1002/bjs.11926 32955109

[40] Yan B Bai DS Zhang C Qian JJ Jin SJ Jiang GQ. Characteristics and risk differences of different tumor sizes on distant metastases of hepatocellular carcinoma: A retrospective cohort study in the seer database. Int J Surg (2020) 80:94–100. doi: 10.1016/j.ijsu.2020.06.018 32619622

[41] Cerban R Ester C Iacob S Grasu M Pâslaru L Dumitru R. Predictive factors of tumor recurrence and survival in patients with hepatocellular carcinoma treated with transarterial chemoembolization. J Gastrointestin Liver Dis (2018) 27(4):409–17. doi: 10.15403/jgld.2014.1121.274.fcr 30574623

[42] Scheiner B Pomej K Kirstein MM Hucke F Finkelmeier F Waidmann O. Prognosis of patients with hepatocellular carcinoma treated with immunotherapy - development and validation of the crafity score. J Hepatol (2022) 76(2):353–63. doi: 10.1016/j.jhep.2021.09.035 34648895

